# Hookworm Disease or Uncinariasis

**Published:** 1912-09

**Authors:** M. H. Boerner

**Affiliations:** Commissioner-in-Charge


### Hookworm Disease or Uncinariasis.—Its Symptoms, Prevalence, and Proposed Methods of Eradication.

M. H. BOERNER, M. D., COMMISSIONER-IN-CHARGE.

In opening the discussion of this subject, the writer earnestly desires to express to the co-editors of this JOURNAL, Dr. F. E. Daniel and his wife, his sincere appreciation and gratitude for this opportunity of acquainting the medical profession and the public in general with the proposed plans of the Hookworm Commission of the Texas State Board of Health. The willing and cheerful offer of these columns is a concrete example of their willingness to aid in improving the sanitary conditions in our State; it is an official endorsement of this public health movement, whose purpose it is to educate the people in physical hygiene, by means of practical demonstrations.

Hookworm disease has undoubtedly been with us for centuries; it is not a new scientific invention to destroy the human race, as many would have you believe, and as many of the more skeptical are firmly asserting, even in face of indisputable proofs. Nor is the stoic the only skeptic; for there is yet another class of millstones to progress—the super-modest and the super-sensitive. False modesty breeds ignorance,—then follows disease. We all, who are born normally, have an intestinal canal, stomach, small and large bowel, and, as long as we live sanely and decently, there is no occasion to parade these useful and hidden internal organs before the external eye. However, when that small man-eater, the hookworm, enters the human body, it never rests until it has fastened onto the lining membrane of the small bowel. Therefore, if we are to discuss hookworm disease, we must think about our inner organs and call them by their proper names and, above all, the public in general must learn that it is no disgrace or shame to have this disease, but it is a depravity to refuse treatment when the infection is known to be present. But to return to the subject—in trying to get the people to cast aside their superabundance of mock-modesty and to think sanely, I am getting the cart before the horse.

In giving the symptoms of hookworm disease, we must start with the entrance of the parasite into the human body, for it begins to cause trouble just as soon as it enters. It is not so kind as some of the disease-producing bacteria, for it does not wait until its children, grand and great-grandchildren are born to help in the deadly struggle. The hookworm is not only energetic in his work of destruction, but it is sly and crafty as well. Think of a worm, microscopical in size, being so wise, so mean, so sneaking as to bore through the skin of the foot or of the leg to gain an entrance, and start on its journey to the small intestine. The old saying goes that the way to a man's heart is through his stomach, but, with the hookworm, the way to the intestine is through the foot. The first symptom of infection, in the vast majority of cases of hookworm, is a burning and itching of the skin of the feet or legs, followed in a few hours by redness, and a

"breaking-out" of "water-blisters." This first stage is commonly known as "ground itch," "dew-poisoning," "toe-itch," etc., and no more thought is given it than to heal up the skin eruption. The little larval form of the hookworm (microscopical in size), after causing this attack of ground itch, passes on down through the layers of the skin, enters the veins and swims in the blood stream through the right chambers of the heart, without exerting the least bit of physical energy. But now, as the blood circulates through the lungs, the trouble begins, for the microscopical worm has grown to be larger than the small capillary blood-vessels of the lungs, hence it is strained out and it is left high and dry in the lung tissues. Not being satisfied with this home, it wriggles its way right through the lung tissues, into the small branches of the "wind-pipe," which it crawls up and enters the throat. At this stage, usually about six or seven weeks after the attack of ground itch, there is a mild sore throat, in fact generally so mild that it is passed unnoticed. The little worm, though now only seven weeks old, is quite wise, for it realizes that, unless it moves on out of the throat, it will be spat out before it can accomplish its deadly purpose. So it slips, unnoticed from the throat, down into the gullet and is swallowed into the stomach. Here it stealthily floats around in our good gastric contents, awaiting the opening of the back-door of the stomach (pylorus), so that it may pass on into the small intestines, its future home. By some sense, whether it is the second or the fifth is not known, it is told that it must "grab a hold"; so, with its strong muscular jaws, it bites into the soft mucous lining of the small bowel, just below the stomach. After so attaching, here it remains, sucking the blood, producing a poison, causing a chronic catarrh (inflammation) for as long a time as ten or twelve years.

When full-grown this parasite is greyish-white, about one inch long and the thickness of a brass pin. Now, with from 100 to 1000 such worms dangling to the inner surface of the bowel, each sapping the blood and interfering with the proper digestion and absorption of the food, what must we expect? Thin blood, pale lips, waxy faces, underdeveloped bodies, slow-thinking minds, dull eyes, backwardness in the school room, "pot-bellies," and many of the multitude of complaints and irregularities grouped under the caption of "indigestion"—that thread-bare cloak of ignorance. Say a child during his fifth summer has several attacks of ground itch, produced by the entrance into the skin of five hundred hookworms, all of which finally reach the small bowel by the circuitous route given above. When this child has reached his eighth year, can you expect his mental and physical development to be on an equal status with that of a healthy child of the same age? Certainly not; for during those three years he has been supporting a family of hookworms, consisting of five hundred members, no one of which was a socialist, every one a parasite, sapping away the child's vitality, rendering him more susceptible to all the acute infectious and contagious diseases, besides thinning his blood, weakening his muscles and dulling his mind. Three-fourths of all the children and young adults who are infected with hookworms are from 3 to 5 years behind their year-age in physical development. These are the "puny children," the "runts," springing from sturdy stock.

The results of the investigation thus far completed show that the infection with hookworm in East Texas ranges from 20 per cent to 75 per cent in the small towns, villages and rural districts. This means that on the average, in many whole counties, 50 per cent of the school children are infected with an intestinal parasite, capable of producing a disease which markedly prevents physical development and seriously retards educational progress. Those heavily infected are physically unable to attend school; the less severely diseased are able to drag along, but their minds are not receptive, nor are they capable of advancing as are normal children.

What is the economic importance of this problem, aside from its humanitarian aspect? Say, in fifty counties in East Texas, there are 250,000 school children, and 25 per cent of these are hookworm sufferers, hence can not attend school, or if they do they are not capable of grasping their full quota of instruction. A yearly school tax of $6.80 is voted for each child of the school age, or $1,700,000. Twenty-five per cent of this, or $425,000, is the annual loss in school appropriations to fifty counties because of hookworm disease. Apply this principle to adults, the producers and the bread-winners. Place the average wage of the farm hand or the mill hand at $1.50 per day; 10,000 adults in each county earning $1.50 per day, but 5 per cent of these are infected with hookworms, and their earning capacity is cut down 5 per cent, aggregating a loss to each county in production of $273,-750, or $13,687,500 is lost annually by fifty counties. Are we going to stand idly by and allow this tremendous amount of human disease and money loss to go on indefinitely, when it is due to an infection that is both easily prevented and safely and surely cured?

To eradicate hookworm disease, we must direct all of our efforts toward three main tasks: (1) determining the exact distribution and degree of infection, (2) getting the present sufferers cured, (3) removing the cause.

Since the Hookworm Commission of the Texas State Board of Health was organized, the private physicians over the entire State have not only heartily endorsed and welcomed the campaign against hookworm disease, but have expressed a willingness to co-operate in every way possible to make the work effective. The writer is indeed grateful to the doctors of Texas for the aid they have given him in determining the distribution of the infection by reporting the number of cases they have treated in their own private practices. Reports sent in by these physicians show that hookworm disease is present in sixty-three counties. The exact percentage of infection will be based upon the results of microscopic examinations of two hundred children of the school age (6 to 18 years), picked at random in each county.

### TREATING THE PRESENT SUFFERERS.

The State Board of Health, working through and with the county health officer, the county board of health and local physicians, establishes in each infected county five dispensaries, where all those applying may receive free examination and treatment. These dispensaries serve a dual purpose; each case cured lessens the dangers of soil pollution by this individual; each cured case is a walking, talking, living advertisement and inducement for the more timid, who are diseased, to seek the much-needed treatment. The drug used is thymol. This treatment is specific, simple, inexpensive and not dangerous when carried out under the direction of a physician. The results, physical and mental improvement, following expulsion of the worms, are little short of a miracle, particularly in children and young adults.

Before a disease can be eradicated, its cause or at least its mode of transmission must be known. The cause of uncinariasis and the mode of transmission of the parasite are definitely known. Each adult female worm, when attached to the small bowel, lays from 1000 to 2000 eggs a day. These eggs pass from the body, thoroughly mixed with the excrement, and each one of these eggs under proper climatic and soil conditions is capable of developing into a microscopical worm in from one to six days. This larva is very energetic, eats everything in sight, grows rapidly and has the power of boring into and through the skin, which is the most common avenue of entrance to the human body. In a small percentage of the cases of hookworm disease the infection is received through the mouth by taking contaminated food and drink, as salad vegetables, berries, water and milk that have been near or in contact with polluted soil. However, the number of cases arising from taking infected food and drink is so small that they may be disregarded in practice. Therefore, to remove the cause of hookworm disease, the people must be encouraged and instructed to improve sanitary environment by being more concerned with careful sewage disposal than with answering the calls of nature in a primitive and neglectful manner.

